# Pregnant Pigs at Slaughter—An Overview of Legal and Ethical Frameworks, Reasons, Occurrence, and Fetal Age Determination

**DOI:** 10.3390/ani16071084

**Published:** 2026-04-01

**Authors:** Frauke Janelt, Johannes Kauffold, Ahmad Hamedy, Katharina Riehn, Philipp Maximilian Rolzhäuser

**Affiliations:** 1 Department Meat Hygiene, Institute of Food Hygiene, Faculty of Veterinary Medicine, Leipzig University, 04103 Leipzig, Germany; 2 Clinic for Ruminants and Swine, Faculty of Veterinary Medicine, Leipzig University, 04103 Leipzig, Germany; 3 Faculty of Life Sciences, Hamburg University of Applied Sciences, 21033 Hamburg, Germany

**Keywords:** animal welfare, culling, fetal age estimation, pregnant sow, slaughter inspection, veterinary legislation

## Abstract

The slaughter of pregnant pigs raises legal, ethical, and animal welfare concerns in pig production. Relevant information for this overview was compiled from research identified through searches of PubMed, Web of Science, and Google Scholar using defined combinations of search terms related to pregnancy, slaughter of sows, fetal age, gestational stage, and prevalence. No lower time limit for publication year was predefined; publications published up to 2025 were considered. Regulations vary widely between countries, with some specifying clear restrictions for animals in late gestation, while many provide no stage-specific limits. Reasons for culling include economic pressures, management practices such as unrecognized pregnancies and mixed-sex housing, and health or welfare issues. In Europe, the prevalence of sows being pregnant at slaughter ranges from 1.5% to 13%, with most fetuses being in the first or second trimester and a small proportion in the final trimester. In Africa, prevalence is higher and more variable, ranging from 9% to 36.14%, with a larger share of fetuses in mid to late gestation. Data from America is limited, reporting prevalences between 5.9% and 13.5%. The comparability of prevalence estimates is limited due to high heterogeneity and differences in study design. Fetal age can be assessed using metric or non-metric methods, applied either *postmortem* or *in vivo* (for example, ultrasonography). Variations in study design, methodology, and population characteristics restrict direct comparability. For legal enforcement and veterinary inspection, reliable fetal age assessment is important, and updated fetometric reference values could contribute to a more consistent interpretation of fetal age.

## Simple Summary

Pregnant pigs are occasionally sent to slaughter for economic, management-related, or health-related reasons, regardless of gestational stage. This raises concerns regarding animal welfare, ethics, and compliance with legal regulations. The presence of pregnant pigs at slaughter, across different fetal developmental stages, underlines why reliable fetal age assessment is necessary for compliance with welfare regulations. Fetal age can be assessed using metric or non-metric methods, applied *postmortem* or *in vivo*, for example, via ultrasonography. Accurate estimation of fetal age is considered an important factor for supporting animal welfare standards and guiding decision-making in pig production.

## 1. Introduction

Within the EU-27’s large-scale pork production system, pig slaughter is a pivotal processing step and accounts for a substantial share of global slaughter volumes. In 2024, approximately 228 million pigs were slaughtered within the European Union (EU) [1], including 44.6 million in Germany [2]. Sows constitute only a small fraction of all slaughtered pigs, and sow-specific slaughter data is rarely reported. In 2024, 600,937 sows were slaughtered in Germany [3]. Modern pig production relies on specialized breeding herds and management strategies aimed at maintaining reproductive performance and herd efficiency. For various reasons, sows are routinely removed (culled) from production, with reduced productivity being one of the major causes [4], and subsequently sent to slaughter. Among these females, some are unintentionally pregnant at different stages of gestation. The slaughter of pregnant sows raises concerns related to sustainability, animal welfare, and ethics. Particularly in advanced gestation, it is associated with welfare-related issues, including the debated potential for fetal perception of pain or distress [5], and it is also widely considered socially unacceptable from an ethical perspective [6]. Legal, ethical, epidemiological, and technical aspects are closely interconnected: regulations influence culling practices, which affect the prevalence of pregnant sows at slaughter, while the methods used to assess fetal age determine how reliably these patterns can be interpreted. Nevertheless, the available literature remains limited and heterogeneous, reflecting differences in study populations, legal frameworks, and methodological approaches across countries. Accordingly, a comprehensive overview of current knowledge is warranted. This overview summarizes prevalence data, legal and ethical considerations, and methods for fetal age determination, highlighting areas where information is limited or heterogeneous.

## 2. Literature Search

A literature search was performed using PubMed, Web of Science, and Google Scholar between May 2023 and January 2026 to identify relevant publications. Searches were conducted using combinations of terms adapted to each database and related to pigs/sows, slaughter, pregnancy and fetuses, prevalence and fetal loss, fetal age, and animal welfare (for details on the search strategy, see Appendix A).

Publications available as full texts and written in English or German were considered. No lower time limit for publication year was predefined; publications published up to 2025 were included.

Additional sources were identified by screening reference lists of retrieved publications and through targeted searches of national publications and compilations (e.g., in review volumes or data summaries).

Grey literature, such as conference proceedings, was included if it had been identified through references in retrieved publications or other sources; no separate search specifically targeting grey literature was performed.

Regulatory texts and guidelines were identified through targeted searches of national and international legal and institutional sources. For regulatory documents not available in English, translations from the original official languages were performed using DeepL Translator (DeepL SE, Cologne, Germany); the possibility of minor inaccuracies or incomplete translations cannot be excluded.

The final selection of studies and reports was performed according to the described criteria. For the identification of prevalence studies, titles and abstracts retrieved through the database searches were screened to identify publications potentially reporting on pregnant pigs/sows at slaughter. Full texts were examined when available to confirm eligibility. In addition, relevant references were identified through screening of key sources and their reference lists, including reports compiling prevalence data, and complemented by studies retrieved through the applied search strategy. Due to the limited number of available studies on this topic, the resulting body of evidence was small, and no discrepancies in study selection arose. Conference abstracts were included when no full-text publications were accessible. No formal risk-of-bias assessment was conducted; however, the limitations of the included studies are discussed in the respective sections.

## 3. Legal and Ethical Frameworks

The slaughter of pregnant pigs is subject to legal regulation, particularly with regard to animal welfare considerations at advanced stages of gestation. Regulatory provisions differ with respect to the gestational stage and their scope of application. At the international level, the World Organisation for Animal Health (WOAH) recommends that animals in advanced gestation should be considered unfit for transport and slaughter [7]. In the EU, Council Regulation (EC) No. 1/2005 specifies that this criterion refers to female animals for which at least 90% of the expected gestation period has elapsed, as well as animals that have given birth within the previous seven days, which are considered unfit for transport [8]. Comparable provisions apply at the national level in, e.g., Switzerland [9] and Croatia [10], while in the United Kingdom, EC No. 1/2005 has been retained in national law following the United Kingdom’s withdrawal from the EU [11]. In Germany, additional national provisions apply. Under the Act on the Prohibition of Trade in Animal Products, the disposal of mammals for the purpose of slaughter during the final third of gestation is prohibited [12]. Sheep and goats are exempt from this prohibition, as the legislator justified this exemption primarily by the predominantly extensive husbandry systems for these species, which often preclude precise recording of breeding dates, complicate reliable pregnancy diagnosis, and limit the practical applicability of established diagnostic methods when compared with pigs [13]. Another exception to the aforementioned law applies regardless of species, covering cases in which culling is required under animal health legislation or justified on veterinary grounds, provided that no overriding animal welfare concerns exist [12]. Taken together, transport regulations in Europe generally prohibit movement of sows during the final 10% of gestation, while Germany additionally enforces trade restrictions for disposal of animals for slaughter. Outside Germany, European countries do not specify further slaughter prohibitions or trade restrictions.

Outside the EU, national legislation on the transport and slaughter of pregnant livestock varies considerably. An overview of relevant regulations is provided in Table 1. Regulatory approaches differ primarily in terms of the stage of gestation, whether the regulatory focus is on transport, slaughter procedures, or both, and the conditions under which exceptions are permitted. Where specific provisions exist, advanced gestation is typically the decisive criterion, with animals in late gestation considered unfit for slaughter, in line with WOAH recommendations [7], as observed in countries such as Canada and Mexico. However, in some countries (e.g., Nigeria and India), even more restrictive regulations have been established, generally prohibiting the slaughter of pregnant females, with exceptions limited to emergency situations, disease control, and veterinary indications. However, based on the available literature and to the best of the authors’ knowledge, most countries worldwide do not explicitly regulate the transport or slaughter of pregnant livestock.

Taken together, regulatory approaches outside Europe are highly heterogeneous. The majority of countries do not specify explicit requirements for the transport or slaughter of pregnant pigs. Among the few exceptions, some African countries impose restrictions on the transport of heavily pregnant animals, whereas explicit slaughter prohibitions are reported only in isolated jurisdictions. In the Americas, regulations for late-gestation animals exist in a limited number of countries, covering both transport and slaughter, while trade restrictions appear absent. In Asia, protective measures during transport are implemented in select countries, and slaughter prohibitions are enforced only in a small number of jurisdictions; trade restrictions are not reported. Overall, transport regulations are more frequently established than explicit slaughter prohibitions, and trade restrictions remain exceptional, highlighting the substantial heterogeneity in legal frameworks and animal protection measures.

The present overview is necessarily limited by the availability of national legislation and regulatory texts. Many documents were accessible only in their original official languages and were translated into English using DeepL Translator (DeepL SE, Cologne, Germany) when no English version was available. As a result, subtle nuances or precise wording in the original texts may not have been fully conveyed, and minor inaccuracies or differences in interpretation cannot be entirely ruled out.

In addition to regulatory aspects, the potential for pain or discomfort in livestock fetuses raises important ethical considerations that warrant scientific scrutiny. It should be emphasized that evidence on fetal pain perception in livestock remains limited and methodologically challenging to obtain, as objective indicators of consciousness and pain in fetuses are inherently difficult to measure and interpret. According to the European Food Safety Authority (EFSA), anatomical and physiological evidence indicates that, during the final third of gestation, livestock fetuses possess the basic structures required for nociception, including sensory afferents, the spinal cord, and partial thalamocortical connections, which may enable the fetus to perceive pain or distress [5]. In pigs, early brain differentiation begins at around 20–22 days of gestation and is followed by progressive cortical development and neuronal migration [29]. The most rapid phase of neural development occurs between approximately 50 days before birth and 40 days after birth, indicating that maturation of central nervous structures continues into the perinatal period [30]. Formation of thalamocortical projections, which are considered relevant for higher sensory processing, has been reported after approximately 56 days of gestation, corresponding to around mid-gestation in pigs [31].

However, the presence of these structures does not necessarily imply conscious pain perception, as cortical activity may be suppressed by neuroinhibitory mechanisms and the low-oxygen intrauterine environment [32]. Experimental observations in pigs further indicate that physiological or motor responses may occur in the absence of conscious perception. For example, fetal movements and convulsive activity have been observed following the death of a sow, particularly in mid- to late-gestation fetuses, and they have been interpreted as manifestations of hypoxic distress mediated primarily by brainstem activation rather than as evidence of conscious pain perception [33].

Studies on human fetuses, however, indicate that measurable physiological responses to noxious stimuli may occur even in the second half of gestation [34]. Some authors have further suggested that pain-like reactions may occur even in the first trimester [35]. It is noteworthy that, similar to humans, maturation of the fetal pig brain continues until the perinatal period [29]. This parallel developmental trajectory may allow cautious comparison between species; however, differences in neurodevelopmental timing and intrauterine physiology mean that the feasibility of extrapolation from human studies to pigs remains limited, and current evidence does not permit the drawing of definitive conclusions regarding fetal pain or discomfort in these species. Building on this assessment, Maas et al. (2022) recommend avoiding the slaughter of pregnant livestock from mid-gestation onwards, except when justified by disease control or emergency considerations [36]. In addition to potential effects on the fetus, transport and slaughter procedures may also affect the pregnant sow, inducing stress during handling and transit, increasing the risk of parturition during transport, and potentially leading to abortion or other health complications [37].

## 4. Reasons for the Slaughter of Pregnant Pigs

To date, no studies have explicitly investigated the reasons for the slaughter of pregnant pigs, and the available information largely stems from observations, expert assessments, and assumptions [5]. Reported and inferred factors can be grouped into three main categories: economic considerations, management-related issues, and animal-health- or welfare-related reasons [5].

In Europe, economic factors include periods of economic crisis, increasing feed and veterinary costs, general market pressure, and production-related considerations. Among the latter, the anabolic effect of pregnancy on carcass weight and muscle yield contributes to heavier carcasses and higher meat yield per animal, which can potentially increase revenue [5]. Management-related factors comprise unrecognized pregnancies due to insufficient supervision, keeping sows together with boars (particularly in outdoor systems), possible contact with wild boars in extensive systems, a lack of systematic pregnancy testing, incomplete or inaccurate documentation of inseminations and pregnancy checks, loss of reproductive information during animal trade, strategic over-insemination to secure planned batch sizes resulting in surplus pregnant animals, and mixed-sex fattening groups containing intact males [5]. Animal health and welfare-related factors include clinical conditions necessitating slaughter, such as lameness, mastitis, and claw disorders, as well as herd depopulation within disease eradication or control programmes (e.g., PRRS) [5].

Outside Europe, information on the slaughter of pregnant pigs is extremely limited. The few available studies originate from African countries and indicate that economic constraints are a major driver, with pigs often serving as a financial reserve and pregnant sows being sold to meet acute monetary needs, irrespective of pregnancy status [38]. Health or performance-related reasons, including primarily reproductive failure, old age, locomotion problems, and disease, have also been reported, but data specific to pregnant pigs remain scarce [39].

Where species-specific data on pigs are lacking, reasons gleaned from other livestock species are informative. Seasonal fluctuations in market demand, including periods associated with cultural or religious festivities, have been reported to influence the timing of slaughter [14]. Management-related factors appear to play a central role, with pregnancies frequently going undetected due to a complete lack of or incomplete pregnancy testing, insufficient breeding records, or loss of reproductive information during trade [40,41,42]. Limited veterinary oversight and poor documentation, particularly in resource-restricted settings, further contribute to these outcomes [43,44].

## 5. Occurrence of Pregnant Pigs at Slaughter

A total of 13 studies reporting the prevalence of pregnant pigs at slaughter were identified across Europe, Africa, and America. In cases where the original studies did not explicitly refer to sows or breeding females, the term “pig” is used here for consistency and clarity. A summary of all reported prevalence data is presented in Table 2. Across the included studies, considerable variation in the prevalence of pregnant pigs at slaughter was observed between countries. In Europe, prevalences generally ranged from approximately 1.5% to 13% [5,39,45,46,47,48]. Finland reported 1.5% [45], Denmark reported 1.6% [46], Sweden reported 2.6% [47], Belgium reported 3% [39], and Germany reported 3% [48], while markedly higher values of up to 13% were reported for Great Britain [49]. In its scientific assessment, the EFSA estimated an overall prevalence of 6% for pigs being pregnant at slaughter in Europe, with approximately 0.5% of animals being in the final trimester [5]. Following the enactment of the Act on the Prohibition of Trade in Animal Products [12], a recent conference contribution by our group demonstrated a prevalence of 0.54% of pregnant sows at slaughter in one of the largest slaughterhouses (processing 10,000 pigs per day) in Germany, based on investigations conducted between 2023 and 2025 [50]. This reduction relative to the previous report for Germany is likely attributable to the enforcement of the Act on the Prohibition of Trade in Animal Products [12] in 2017.

Outside Europe, prevalences were generally higher and more variable. In Africa, reported values ranged from 9% [38] to 36.1% [58] in Nigeria, despite general prohibitions, and 15.1% [54] to 29.3% [57] in Ghana, while a prevalence of 18% was reported for Cameroon [55]. In North and South America, prevalences of 5.9% in the United States [59] and 13.5% in Venezuela [60] have been reported.

The prevalence studies included in this overview exhibit substantial heterogeneity, which limits the interpretability of the data. Determining the prevalence of pregnant pigs was not the primary objective in [39,45,46,47,49,55,59,60], whereas six studies [38,48,50,54,57,58] explicitly aimed to measure prevalence. Among the latter, only two studies [48,50] clearly specified the slaughter class as sows; the remaining prevalence studies reported on “pigs” without further classification. Sample sizes varied widely, with particularly small numbers in [39,47,49,54,55,60], limiting the precision of prevalence estimates. Gestational age determination was explicitly conducted in [38,48,49,50,54,57], whereas other studies did not report this information, leading to a lack of important data on gestational age distribution. Study periods ranged considerably, from a few months to several years, with the absolute number of animals investigated varying independently of study duration. These methodological differences, heterogeneity in study focus, limited sample sizes, incomplete gestational age assessment, and inconsistencies in reporting relevant data contribute to uncertainty in prevalence estimation and should be taken into account when interpreting the findings.

## 6. Determination of Fetal Age in Pigs

Fetal age estimation in pigs is essential for assessing compliance with established gestational thresholds. For an average gestation length of 114 days, the threshold corresponding to 90% of gestation (EC No. 1/2005 [8]) occurs at approximately day 103, and that corresponding to the final third of gestation (Act on the Prohibition of Trade in Animal Products, Germany [12]) occurs at approximately day 77. The transition from the first to the second trimester occurs around day 38 of gestation.

### 6.1. Gestational Age Distribution for Slaughtered Pregnant Pigs

Among the studies reporting prevalences of slaughtered pregnant pigs, six additionally provided information on fetal age, allowing an assessment of gestational stage distribution (Table 2) [38,48,49,50,54,57,58]. In these studies, fetal age determination was performed using one of the methods described above.

Within Europe, the majority of fetuses were found in the first or second trimester. In Germany, earlier assessments indicated that 47%, 44%, and 9% were found in the first, second, and third trimesters, respectively, with an overall prevalence of 3% [48]. More recent data reveal an overall prevalence of 0.54%, with 21.4%, 71.4%, and 7.1% in the first, second, and third trimesters, respectively [50]. In Great Britain, 57.9%, 21.1%, and 21.1% were in the first, second, and third trimesters, with an overall prevalence of 13% [49].

Outside Europe, distributions were shifted toward later gestation. In Nigeria, 59.7%, 29.6%, and 10.6% were in the first, second, and third trimesters [38], whereas in Ghana, two independent studies reported values of 16.7%, 61.4%, and 21.9% for the first, second, and third trimesters [54] and 16.5%, 43.2%, and 40.3% for the first, second, and third trimesters [57], respectively. Differences in fetal age distributions across studies reflect country-specific patterns; however, direct comparability between populations is limited by variations in study design, sample size, and the methods used for fetal age determination.

### 6.2. Methods for Age Determination

In swine production, gestational age in sows is commonly estimated based on breeding records. However, in cases where information on gestational age is unavailable, fetal age can be assessed *postmortem* or *in vivo*, including via ultrasonography. *Postmortem* assessment may occur either unintentionally following routine slaughter, wherein fetuses are examined as part of standard *postmortem* investigations, or for research purposes, wherein fetal age is determined deliberately in a study setting. Measurement methods for fetal age determination in pigs are broadly classified as metric and non-metric.

Among metric approaches applied *postmortem*, crown–rump length (CRL) is the most widely applied measurement for determining fetal age in Europe. Schnorr and Kressin (2011) [61], based on historical data derived from Habermehl (1975) [51] and Evans and Sack (1973) [52], classified fetuses into trimester boundaries (approximately days 40 and 80, rounded values) using CRL thresholds of 5 cm and 21 cm. As for day 40 of gestation, similar values have been reported by McGeady et al. (2017) [62], with 4.5 cm, and by Knight et al. (1977) [63], with 5.1 cm. For day 80, Knight et al. (1977) [63] documented a CRL of 19.2 cm. At around day 100 of gestation, corresponding to 90% of gestation, Schnorr and Kressin (2011) [61] described a CRL of approximately 27 cm, in agreement with McGeady et al.’s assessment (2017) [62]. A summary of these CRL measurements and corresponding references is provided in Table 3.

In addition to direct CRL measurement, Odlaug (1955) [53] proposed a mathematical approach for estimating fetal age based on CRL, expressed as Age (days) = 3 × (y + 21), where y represents crown–rump length in centimeters, 3 is a species-specific growth factor for pigs, and 21 corresponds to the approximate embryonic–fetal transition. This formula was applied to *postmortem* fetuses in subsequent investigations, including studies on African pig populations [38,54], and provided a calculated estimate of fetal age rather than relying on predefined trimester thresholds. Beyond CRL, cranial length (rostro-occipital distance) has also been used as a metric parameter, ranging from 6.1 to 6.7 cm in pig fetuses between days 76 and 80 of gestation [64].

A combined approach to estimating fetal age has been proposed by Wrathall et al. (1974) [56], using radiographic examination to integrate metric measurements of diaphyseal length with assessment of ossification in the fore- and hindlimb bones. Using this method, the authors determined the diaphyseal length of, e.g., the humerus to be approximately 0.099 cm on day 40, 2.08 cm on day 80, and 3.29 cm on day 100 of gestation. Corresponding ossification scores (a semi-quantitative index reflecting the overall extent of bone ossification) were approximately 6.89 on day 40, 43.1 on day 80, and 71.9 on day 100 of gestation. While these scores provide an indication of developmental progression, individual ossification centers were not always clearly identifiable, and age cannot be reliably determined on the basis of ossification alone; combining these observations with metric measurements of limb length improved accuracy.

As a non-metric method, fetal age in pigs can also be estimated based on developmental features such as eyelid formation, tooth growth, and fetal weight [61,65,66] as well as organ development and organ weight [67].

However, many of the classical reference values are derived from historical datasets in which breeding dates, breed information, sample size, or methodological details were not consistently reported. Although some studies [67,68,69] included known gestational ages, the lack of comparison with known breeding dates in most studies represents an important limitation. Differences in study design, genetic background, and timing of measurements may further limit direct comparability between studies. Measurement variability, including potential inter-observer differences, could also contribute to uncertainty. These factors should be kept in mind when interpreting fetal age data.

### 6.3. New Approaches to Fetal Age Determination

The applicability of previously reported fetal age data is increasingly uncertain. Many earlier studies were based on *postmortem* examinations and often lack detailed information on breed, genetic background, or measurement techniques, limiting comparability. In addition, genetic progress has increased litter sizes and altered fetal growth dynamics; smaller fetuses within larger litters may develop more slowly, complicating direct comparisons with historical data [70,71,72]. These limitations indicate the need for updated approaches to fetal age determination.

*In vivo* ultrasonographic assessment, as described by Kähn et al. (2004) [73], provides a suitable alternative, as it enables evaluation of fetal development in living animals and avoids the need to cull pregnant sows for research purposes. In commercial swine production, *in vivo* ultrasonography is routinely used for pregnancy detection from approximately days 20 to 25 [74,75] post-insemination. Building on this concept, an ultrasound-based approach to fetometric age determination in swine across genotypes differing in prolificacy has been described [76]. Seventy pregnancies from low (purebred German Saddlelback)- and medium-to-high-level prolific (purebred German Landrace and Duroc × German Landrace hybrids) lines were repeatedly examined using transabdominal ultrasonography. Clearly identifiable and practically applicable fetometric parameters—including rostro-occipital distance (ROD), biparietal diameter (BPD), orbital diameter (OD), sternum length (SL), thorax diameter (TD), CRL, and body diameter (BD)—were recorded. Gestational age was assigned based on documented insemination dates. In addition, a validation of the *in vivo* measurements demonstrated their comparability with *postmortem* assessments based on comparative sonographic and subsequent *postmortem* measurements in gravid uteri from a slaughterhouse [76].

Crown–rump length served as the primary parameter for fetal age estimation, with thresholds of ≥3.2 cm and ≥16.3 cm indicating the transition to the second and third trimesters, respectively. Additional fetometric parameters appeared to improve estimation in late gestation, particularly after approximately 90% of the gestation period has elapsed. Relative to data from earlier studies, fetuses from contemporary hybrid production systems tended to be smaller at comparable gestational stages (Table 3). These observations suggest that growth patterns in modern pig populations may differ to some extent from those reflected in historically cited CRL thresholds (e.g., approximately 5 cm and 21 cm for trimester boundaries [51,52]). Consequently, the use of updated ultrasound-derived reference values may lead to the classification of some fetuses into slightly different gestational stages compared with historical datasets, particularly within the final trimester However, the interpretation of fetometric thresholds in regulatory contexts should be approached cautiously. Although the ultrasound-based measurements were validated against *postmortem* assessments in the cited study [76], the currently available reference data derive from a limited number of genotypes and management conditions. Large-scale, multi-site validation across a broader range of pig populations and production systems has not yet been performed. Therefore, broader external validation, including larger datasets and diverse production environments, would be necessary before such approaches could be considered for wider methodological or regulatory application.

## 7. Conclusions

Pregnant pigs at slaughter have been documented in multiple countries and across continents, with reported prevalence varying widely. Fetal age can be assessed using metric and non-metric approaches; while historical studies primarily relied on *postmortem* measurements, more recent methods for research purposes have increasingly employed *in vivo* ultrasonography. Differences in methodology, study design, and population characteristics restrict direct comparison of data across studies and regions and represent a limitation of this overview. The lack of data, together with insufficiently described methodologies, on the occurrence of pregnant-pig slaughtering and assumptions regarding the reasons for slaughtering pregnant pigs may limit the scope and conclusions of this overview. These observations highlight the need for further studies to address these issues within the context of the slaughter of pregnant pigs. Overall, this review emphasizes variability in prevalence, methodological approaches, and the emerging role of *in vivo* ultrasonography while underscoring the importance of cautious interpretation and additional standardized research.

## Figures and Tables

**Table 1:** Regulations on the transport and slaughter of pregnant livestock outside Europe.

Continent	Country/Region	Transport Regulation	Slaughter Regulation	Source
Africa	Nigeria	—	Slaughter of pregnant animals is prohibited except in emergencies or with veterinary supervision	[14]
Africa	Tanzania	Transport is allowed only for disease control or to alleviate suffering; it requires pregnancy diagnosis by a veterinarian	Slaughter is allowed only for disease control or to alleviate suffering	[15]
Africa	Algeria	—	Slaughter of pregnant females is allowed only with official veterinary authorization	[16]
Africa	Kenya	Heavily pregnant animals are classified as unfit for transport; exceptions are made only with veterinary authorization or in emergencies	—	[17]
Africa	South Africa	Heavily pregnant animals are classified as unfit for transport; exceptions are made only with veterinary authorization or in emergencies	—	[18,19].
America	Canada	Animals in late gestation or immediately postpartum are considered unfit for transport	Animals in late gestation or immediately postpartum are considered unfit for slaughter	[20]
America	Mexico	Late-gestation animals are considered unfit for transport	Late-gestation animals are considered unfit for slaughter	[21,22]
America	Uruguay	Late-gestation animals are considered unfit for transport	Late-gestation animals are considered unfit for slaughter	[23]
Asia	India	—	Slaughter is prohibited for pregnant animals or those with dependent offspring	[24]
Asia	Pakistan	—	Animals that have completed 1/3 of their pregnancy must not be slaughtered	[25,26,27]
Asia	South Korea	Protective measures must be taken during transport of pregnant or lactating animals	—	[28]
International recommendation	World Organisation for Animal Health (WOAH)	Animals in advanced gestation are considered unfit for transport except in emergency or disease-control situations	Animals in advanced gestation are considered unfit for slaughter except in emergency or disease control situations	[7]

**Table 2:** Reported occurrence of pregnant pigs at slaughter worldwide.

Prevalence	Country	Number of Female Pigs Slaughtered	Gestational Age	Age Determination Approach	Period	Source
Europe						
0.54%	Germany	2597	1. T: 21.4% 2. T: 71.4% 3. T: 7.1%	Habermehl (1975) [51] and Evans and Sack (1973) [52]	December 2023–April 2025	[50]
1.5%	Finland	1708		None	October 1992–September 1993	[45]
1.6%	Denmark	2989		None	February–November 2008	[46]
2.6%	Sweden	115		None	August 1993–October 1994	[47]
almost 3%	Belgium	502		None	December 2010–April 2011, December–February 2012	[39]
3%	Germany	11,137	1. T: 47% 2. T: 44% 3. T: 9%	Habermehl (1975) [51] and Evans and Sack (1973) [52]	May–October 2017	[48]
13%	Great Britain	142	1. T: 57.9% 2. T. 21.1% 3. T. 21.1%	Requested insemination date	2006–2008	[49]
Africa						
9% Min.: 6.67% Max.: 12.50%	Nigeria	1265	1. T: 59.7% 2. T: 29.6% 3. T: 10.6%	Odlaug (1955) [53]	September–December 2012	[38]
15.1%	Ghana	870	1. T: 16.7% 2. T: 61.4% 3. T: 21.9%	Odlaug (1955) [53]	February–April 2018	[54]
18%	Cameroon	450		None		[55]
29.27%	Ghana	1739	1. T: 16.53% 2. T: 43.17% 3. T: 40.34%	Wrathall et al. (1974) [56]	November 2014–March 2015	[57]
36.14%	Nigeria	1203		None	October–December 2000–2007	[58]
America						
5.9%	USA	3158		None	April–September 2005	[59]
13.5%	Venezuela	244		None	April–November 2009	[60]

T = trimester.

**Table 3:** Summary of crown–rump length (CRL) values in *postmortem* pig fetuses.

Gestation Day	Crown–Rump Length (cm)	References
40	4.5–5.1	[51,52,61,62,63]
80	19.2–21.0	[61,63]
100	27.0	[61,62]

## Data Availability

No new data were created or analyzed in this study. Data sharing is not applicable to this article.

## Appendix A

**Table A1:** Search strategy.

Database	Search Strategy
PubMed	(“sow”[tiab] OR “swine”[tiab] OR “pig”[tiab]) AND (“slaughter”[tiab] OR “slaughterhouse”[tiab] OR “abattoir”[tiab]) AND ((“pregnancy”[tiab] OR “gestation”[tiab] OR “fetus”[tiab] OR “foetus”[tiab]) OR (“prevalence”[tiab] OR “foetal wastage”[tiab]) OR (“age determination”[tiab] OR “gestational stage”[tiab] OR “fetal age”[tiab]) OR (“animal welfare”[tiab] OR “fetal pain”[tiab] OR “fetal perception”[tiab]))
Web of Science	TS=(“sow” OR “swine” OR “pig”) AND TS=(“slaughter” OR “slaughterhouse” OR “abattoir”) AND (TS=(“pregnancy” OR “gestation” OR “fetus” OR “foetus”) OR TS=(“prevalence” OR “foetal wastage”) OR TS=(“age determination” OR “gestational stage” OR “fetal age”) OR TS=(“animal welfare” OR “fetal pain” OR “fetal perception”))
Google Scholar	(“sow” OR “swine” OR “pig” OR “Sau” OR “Schwein”) AND (“slaughter” OR “slaughterhouse” OR “abattoir” OR “Schlachtung” OR “Schlachthof”) AND ((“pregnancy” OR “gestation” OR “fetus” OR “foetus” OR “Trächtigkeit”) OR (“prevalence” OR “foetal wastage” OR “Prävalenz”) OR (“age determination” OR “gestational stage” OR “fetal age” OR “Altersbestimmung” OR “Trächtigkeitsstadium”) OR (“animal welfare” OR “fetal pain” OR “fetal perception” OR “Tierwohl”))
